# Correction: Fang et al. Gandouling Mitigates CuSO_4_-Induced Heart Injury in Rats. *Animals* 2022, *12*, 2703

**DOI:** 10.3390/ani13223527

**Published:** 2023-11-15

**Authors:** Shuzhen Fang, Wenming Yang, Kangyi Zhang, Chuanyi Peng

**Affiliations:** 1 University Hospital, Anhui Agricultural University, 130 Changjiang Road West, Shushan District, Hefei 230036, China; fangshuzhen@ahau.edu.cn; 2 Department of Neurology, The First Affiliated Hospital of Anhui University of Chinese Medicine, 117 Meishan Road, Shushan District, Hefei 230031, China; 3 School of Tea and Food Science & Technology, Anhui Agricultural University, 130 Changjiang Road West, Shushan District, Hefei 230036, China; zhky202204@163.com (K.Z.); pcy0917@ahau.edu.cn (C.P.)

## Error in Figure

In the original publication [1], there was a mistake in ***Figure 4*** as published. ***Figure 4F*** was unintentionally duplicated, which was a careless mistake during the revision. Here is the new ***Figure 4*** that has been corrected. The corrected ***Figure 4*** appears below. The authors state that the scientific conclusions are unaffected. This correction was approved by the Academic Editor. The original publication has also been updated. We thank an astute reader for their input on this matter.

The authors would like to apologise for any inconvenience caused.

## Figures and Tables

**Figure 4:**
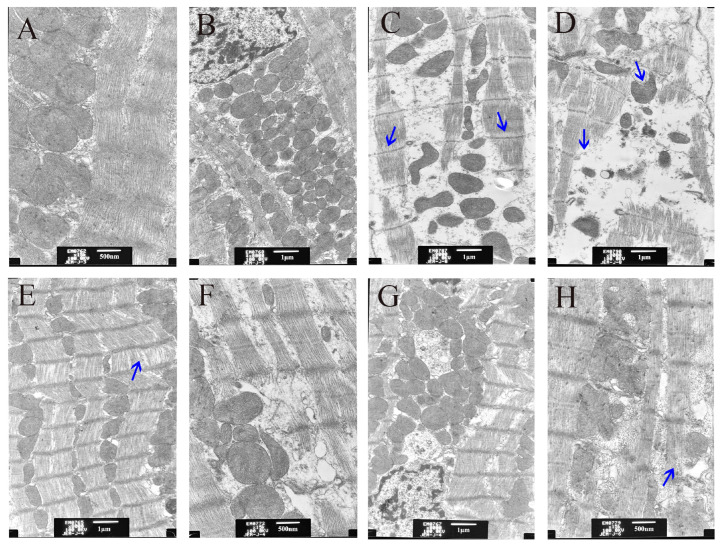
Ultrastructure of the rat heart tissue after different treatments under electron microscope. (**A**,**B**) Control; (**C**,**D**) CuSO_4_; (**E**,**F**) GDL + CuSO_4_; (**G**,**H**) Penicillamine + CuSO_4_. The arrows indicated the ultrastructural changes after CuSO_4_ exposure, GDL and penicillamine treatments.
